# Supplementary material for: ProteinShader: illustrative rendering of macromolecules
Source: BMC Struct Biol. 2009 Mar 30;9:19. doi: 10.1186/1472-6807-9-19 (PMC2672931; doi:10.1186/1472-6807-9-19)
Supplement: Additional file 1 — ProteinShader program without source code. This compressed file contains the complete ProteinShader program including associated libraries, but no source code. A README.txt file gives an overview of the ProteinShader distribution, and the index.html file in the help subdirectory has directions on getting started with the program as well as a set of tutorials. [file 1472-6807-9-19-S1.zip › ProteinShader-beta-0_9_4-binary/help/api/org/proteinshader/graphics/typography/GlyphSet.html]

GlyphSet (ProteinShader API)


|  |  |  |
| --- | --- | --- |
| **Overview** \| **Package** \| **Class** \| **Use** \| **Tree** \| **Deprecated** \| **Index** \| **Help** | |  |
| **PREV CLASS**   **NEXT CLASS** | **FRAMES**    **NO FRAMES**     **All Classes** |  |
| SUMMARY: NESTED \| FIELD \| CONSTR \| METHOD | DETAIL: FIELD \| CONSTR \| METHOD |  |


---


## org.proteinshader.graphics.typography Class GlyphSet

```
java.lang.Object
  org.proteinshader.graphics.typography.GlyphSet
```

---

``` public class GlyphSet extends Object ```

Stores a set of Glyph objects.

---

**Constructor Summary**

`GlyphSet(String filename,
String fontname,
String typeface,
int fontSize,
int bytesPerPixel,
int format,
int dataType)`
  
          Constructs a GlyphSet.


| **Method Summary** | |
| --- | --- |
| `void` | `addGlyph(Glyph glyph)`             Adds a Glyph object to this GlyphSet. |
| `int` | `getBytesPerPixel()`             Returns the number of bytes per pixel. |
| `int` | `getDataType()`             Returns the OpenGL data type (such as GL.GL\_UNSIGNED\_BYTE). |
| `String` | `getFilename()`             Returns the name of the file the glyphs were read from. |
| `String` | `getFontname()`             Returns the name of the font this set of glyphs belongs to. |
| `int` | `getFontSize()`             Returns the font size. |
| `int` | `getFormat()`             Returns the OpenGL format (such as GL.GL\_LUMINANCE\_ALPHA). |
| `Glyph` | `getGlyph(String name)`             Returns the Glyph object for the name given as an argument (or null if it cannot be found). |
| `List<Glyph>` | `getGlyphs(String text)`             Returns a list of Glyph objects corresponding to the characters of the String given as an argument. |
| `String` | `getTypeface()`             Returns the typeface for the glyphs (bold, italic, regular, *etc*.). |
| `String` | `getUnknownChars()`             Returns a String composed of any characters that were missing (no corresponding Glyph) when the last call to getGlyphs(text) was made. |
| `boolean` | `hadUnknownChars()`             Returns true if the last call to getGlyphs(text) had any unknown characters in the String (unknown in the sense that the GlyphSet did not have a Glyph for a character). |
| `Iterator<Glyph>` | `iteratorGlyphs()`             Returns an Iterator for the Glyphs. |
| `int` | `numberOfGlyphs()`             Returns the number of Glyphs in the set. |

**Methods inherited from class java.lang.Object**

`clone, equals, finalize, getClass, hashCode, notify, notifyAll, toString, wait, wait, wait`

**Constructor Detail**

### GlyphSet

```
public GlyphSet(String filename,
                String fontname,
                String typeface,
                int fontSize,
                int bytesPerPixel,
                int format,
                int dataType)
```

:   Constructs a GlyphSet.

    **Parameters:**: `filename` - the name of the file the glyph set was read from.: `fontname` - the name of the font the glyphs belong to.: `typeface` - a type such as bold, italic, or regular.: `fontSize` - the size of the font in points.: `bytesPerPixel` - there can be 1 to 4 bytes per pixel.: `format` - the OpenGL format (such as GL.GL\_LUMINANCE\_ALPHA).: `dataType` - the OpenGL data type (such as GL.GL\_UNSIGNED\_BYTES).


**Method Detail**

### addGlyph

```
public void addGlyph(Glyph glyph)
```

:   Adds a Glyph object to this GlyphSet.

    :   **Parameters:**: `glyph` - the Glyph to add.

---


### getGlyph

```
public Glyph getGlyph(String name)
```

:   Returns the Glyph object for the name given as an argument (or
    null if it cannot be found).

    :   **Parameters:**: `name` - the name of a glyph is usually a single character.

---


### getGlyphs

```
public List<Glyph> getGlyphs(String text)
```

:   Returns a list of Glyph objects corresponding to the characters
    of the String given as an argument.

---


### getUnknownChars

```
public String getUnknownChars()
```

:   Returns a String composed of any characters that were missing (no
    corresponding Glyph) when the last call to getGlyphs(text) was
    made.

---


### hadUnknownChars

```
public boolean hadUnknownChars()
```

:   Returns true if the last call to getGlyphs(text) had any unknown
    characters in the String (unknown in the sense that the GlyphSet
    did not have a Glyph for a character).

    :   **Returns:**: Boolean to indicate if there were unknown characters in the last call to getGlyphs(text).

---


### getFilename

```
public String getFilename()
```

:   Returns the name of the file the glyphs were read from.

    :   **Returns:**: The filename as a String.

---


### getFontname

```
public String getFontname()
```

:   Returns the name of the font this set of glyphs belongs to.

    :   **Returns:**: The fontname as a String.

---


### getTypeface

```
public String getTypeface()
```

:   Returns the typeface for the glyphs (bold, italic, regular,
    *etc*.).

    :   **Returns:**: The typeface as a String.

---


### getFontSize

```
public int getFontSize()
```

:   Returns the font size.

    :   **Returns:**: The font size as an integer.

---


### getBytesPerPixel

```
public int getBytesPerPixel()
```

:   Returns the number of bytes per pixel.

    :   **Returns:**: The bytes per pixel as an integer.

---


### getFormat

```
public int getFormat()
```

:   Returns the OpenGL format (such as GL.GL\_LUMINANCE\_ALPHA).

    :   **Returns:**: The format as an integer.

---


### getDataType

```
public int getDataType()
```

:   Returns the OpenGL data type (such as GL.GL\_UNSIGNED\_BYTE).

    :   **Returns:**: The data type as an integer.

---


### iteratorGlyphs

```
public Iterator<Glyph> iteratorGlyphs()
```

:   Returns an Iterator for the Glyphs.

    :   **Returns:**: An Iterator for the Glyphs.

---


### numberOfGlyphs

```
public int numberOfGlyphs()
```

:   Returns the number of Glyphs in the set.

    :   **Returns:**: The total number of Glyphs in the set.


---


|  |  |  |
| --- | --- | --- |
| **Overview** \| **Package** \| **Class** \| **Use** \| **Tree** \| **Deprecated** \| **Index** \| **Help** | |  |
| **PREV CLASS**   **NEXT CLASS** | **FRAMES**    **NO FRAMES**     **All Classes** |  |
| SUMMARY: NESTED \| FIELD \| CONSTR \| METHOD | DETAIL: FIELD \| CONSTR \| METHOD |  |


---

# *Copyright © 2007-2008*
